# Green Synthesis of Ag/Ag_2_O Nanoparticles Using Aqueous Leaf Extract of *Eupatorium odoratum* and Its Antimicrobial and Mosquito Larvicidal Activities

**DOI:** 10.3390/molecules22050674

**Published:** 2017-04-28

**Authors:** Elias E. Elemike, Damian C. Onwudiwe, Anthony C. Ekennia, Christopher U. Sonde, Richard C. Ehiri

**Affiliations:** 1 Material Science Innovation and Modelling (MaSIM) Research Focus Area, Faculty of Agriculture, Science and Technology, North-West University (Mafikeng Campus), Private Bag X2046, Mmabatho 2357, South Africa; chemphilips@yahoo.com; 2 Department of Chemistry, School of Mathematical and Physical Sciences, North-West University (Mafikeng Campus), Mmabatho 2735, South Africa; 3 Department of Chemistry, College of Sciences, Federal University of Petroleum Resources Effurun, Delta State, Nigeria; 4 Department of Chemistry, Federal University, Ndufu-Alike Ikwo (FUNAI), P.M.B. 1010, Abakaliki, Ebonyi State, Nigeria; chemisttony@gmail.com (A.C.E.); xtopheraries@yahoo.co.uk (C.U.S.); richcee2003@yahoo.com (R.C.E.)

**Keywords:** Nanoparticles, silver, larvicidal efficacy, antimicrobial, *Eupatorium odoratum*

## Abstract

The health challenges associated with pathogens and ectoparasites highlight the need for effective control approaches. Metal nanoparticles have been proposed as highly effective tools towards combatting different microbial organisms and parasites. The present work reports the antimicrobial and larvicidal potential of biosynthesized Ag/Ag_2_O nanoparticles using aqueous leaf extract of *Eupatorium odoratum* (EO). The constituents of the leaf extract act as both reducing and stabilizing agents. The UV-VIS spectra of the nanoparticles showed surface plasmon resonance. The particle size and shape of the nanoparticles was analysed by transmission electron microscopy (TEM). The larvicidal study was carried out using third and fourth instar *Culex quinquefasciatus* larvae. The mosquito larvae were exposed to varying concentrations of plant extract (EO) and the synthesized nanoparticles, and their percentage of mortality was accounted for at different time intervals of 12 h and 24 h periods of exposure. The nanoparticles were more lethal against third and fourth instars of *Culex quinquefasciatus* larvae at the 24 h period of exposure with lower lethal concentration values (LC50 = 95.9 ppm; LC90 = 337.5 ppm) and (LC50 = 166.4 ppm; LC90 = 438.7 ppm) compared to the plant extract (LC50 = 396.8 ppm; LC90 = 716.8 ppm and LC50 = 448.3 ppm; LC90 = 803.9 ppm, respectively). The antimicrobial properties of the nanoparticles were established against different clinically-isolated microbial strains and compared to that of the plant extract (EO) and standard antimicrobial drugs. The nanoparticles were generally more active than the plant extract against the selected microbial organisms. The Gram-negative bacterial strains *Escheerichua coli* and *Salmonella typhi* were more susceptible towards the nanoparticles compared to the Gram-positive strains and the fungal organism.

## 1. Introduction

Synthesis of nanoparticles for applications in areas such as medicine, catalysis, environment and biotechnology has become an area of interest [1,2]. Nanoparticles have been prepared by physical, chemical, electrochemical, sonochemical, irradiation, and biological routes. However, the biological route is most preferred and it includes microbial nanosynthesis (use of microrganisms) and phytonanosynthesis (use of plants) approaches. Microbial nanosynthesis takes a long period of time to obtain adequate synthetic biomass and also the same biomass cannot be reused for another synthesis [3]. The use of plant sources in nanoparticle synthesis is a more preferred route due to a shorter period of synthesis and also due to the fact that most plant sources satisfy the roles of stabilising, reducing, and capping effects, thereby limiting the amount of raw materials and promoting green technologies [4]. Other advantages of phytonanotechnology include the safer nature of synthesis, biocompatibility, non-toxic nature, cost-effectiveness, sustainability, and environmental friendliness, as well as a lack of underlined special culture preparation and isolation techniques [5,6]. In addition, biosynthesis involving the use of plant extracts usually occurs in aqueous medium, which is cheap and offers no limitations in terms of applications.

Conversely, the use of plant extracts in nanoparticle synthesis can change the oxidation state of metal ions through some perceived metabolic processes. By so doing, energy is conserved when the oxidized metal ions act as terminal electron acceptors and the capping effect by the active components from the plant substrate leads to modified characteristics of the nano-formed metal [7]. However, not all biological entities can synthesize nanoparticles due to their enzyme activities and intrinsic metabolic processes, hence, selection of the biological medium entails versatile screening procedures and careful selection of an appropriate biological entity that can produce nanoparticles with well-defined properties, such as size and morphology [8,9,10].

The focus of green chemistry is to design chemical products and processes that reduce or eliminate the use or generation of hazardous substances that affects human health and the environment and, in that context, we tend to develop nanoparticles that would not only clean up vector breeding environments, but also with antimicrobial potentials. Vector control is an important aspect of public health mostly with respect to mosquito-borne diseases. Several methods have been implored to prevent the proliferation of mosquito-borne diseases. Most common of them is the application of synthetic insecticides, such as organochlorine and organophosphate compounds on mosquito-infested areas [11]. However, the introduction of chemically-based insecticides in the environment poses some health challenges due to its toxic and non-degradable compositions. Additionally, in many instances, resistivity of mosquitoes to these common insecticides has been reported [12]. Insecticidal residues enter into the ecosystem, through the food web, circulates, and biomagnifies. With several environmental protection acts on the application of chemicals in the natural environment, different governments’ agencies across the globe are interested in studieswhich are inclined towards making the environment a safer place devoid of harmful chemicals [13]. This has stimulated several studies on natural insecticides as an alternative control measures, focusing on plant-derived compounds, including volatile chemical constituents (essential oils), as potentially bioactive substances against mosquito larvae [14]. Identification, isolation, and mass synthesis of bioactive compounds against mosquitoes are imperative for the management of mosquito-borne diseases. 

Similarly, the incorporation of these bioactive plant components on nanoparticles has been investigated as potential alternatives to chemical insecticides [15,16,17]. The use of nanoparticles in the control of mosquito-borne diseases presents many advantages because it is eco-friendly, non-hazardous, cost effective, has greater surface area, and is also biocompatible [18]. Larviciding is the application of chemicals to kill mosquito larvae or pupae in the water. The control of mosquitoes’ larvae usually involves the use of dichlorodiphenyltrichloroethane (DDT), organophosphate temephos, methoprene, pyrethroids, phytochemicals, and soil bacteria (*Bacillus thuringiensis israelensis* and *Bacillus sphaericus*) [19]. Recently, different researchers have reported larvicidal activity of green synthesized nanoparticles. Silver NPs of different plant extracts, such as *Feronia elephantum* [20], *Azadirachta indica* (Neem) [21], *Morinda tinctoria* [22], *Agave sisalana* [23], *Eclipta prostrata* [24], *Ficus racemosa* [25], and *Sterculia foetida* L. [26] have been reported against different stages of larvae of *Culex quinquefasciatus*, *Anophelesstephensi*, and *Aedes aegypti*. The nanoparticles have been reported to possess larvicidal activity, which in most cases, exhibited better larvicidal properties than their precursor plant extracts. It is also generally more effective and target-specific than applying chemicals to kill adult mosquitoes (adulticiding).

*Eupatorium odoratum* (otherwise called *Chromolaena odoratum*) is a shrub of the sunflower family (*Asteraceae*). It is native in Central and South America and distributed all over tropical Asia, Western Africa, and in parts of Australia, including subtropical areas of the world [27]. *E. odoratum* is used traditionally for the treatment of wounds, skin infections, inflammation, insect repellents, etc. [28,29]. Furthermore, the leaf extract possess antioxidant, antimicrobial, antigonorrhoeal, antipyretic, antisplasmodic, diuretic, anti-inflammatory, and analgesic properties [30,31,32,33,34,35,36]. Proximate analysis carried out on the leaf of *E. odoratum* revealed 16.20% crude protein (with histidine and phenylalanine being very high), 50.82% total carbohydrate, 26.57% crude fibre, and 6.17% total ash by dry weight. It also contains wide varieties of secondary metabolites, which include tannins, saponins, phytates, flavonoids, betacyanins, alkaloids, steroids, terpenoids, phenols, quinones, and glycosides [37]. Similarly, an oil extract from *E. odoratum* leaf was reported to contain pregeijerene, epi-cubebol, cubebol, cis-sabinene hydrate, germacrene-D-4-ol, germacrene D, geijerene, cyperene, α-muurolol, khusimone, β-copaen-4α-ol, camphor, limonene, vestitenone, bulnesol, and trans-ocimene [38]. Oil obtained from other parts of the plant like aerial part and flowers were dominated by phytochemicals, like monoterpene and sesquiterpene hydrocarbons [39,40,41,42,43]. 

In continuation of our research on the biological potentials of green synthesized silver nanoparticles [44,45], we investigate herein, the mosquito larvicidal efficacy and antimicrobial potentials of silver/silver oxide nanoparticles synthesized by the use of leaf extract of *E. odoratum*. The formation of the nanoparticles was monitored by the use of UV-VIS spectroscopy while the size and shape of the nanoparticles were established using transmission electron microscopy (TEM). 

## 2. Materials and Methods

### 2.1. Plant Collection

*E. odoratum* leaves were collected from Warri in Delta State Nigeria and generically identified by a taxonomist. The leaves were washed several times with tap water to remove dust and soil. They were subsequently washed with distilled water and dried in the shade at room temperature and stored at 4 °C for further use.

### 2.2. Preparation of Aqueous Leaf Extract of E. odoratum

The dried leaves of *E. odoratum* were ground into powder form using an electrical stainless steel blender [19,22]. The powder was mixed with 250 mL distilled water and boiled for 1 h. The extract was filtered through Whatman #1 filter paper to obtain a clear solution. The filtrate was used immediately for nanoparticle synthesis.

### 2.3. Synthesis of Silver/Silver Oxide Nanoparticles

The nanoparticles were prepared at different concentration ratios of the plant extract to the AgNO_3_ according to other reported methodologies [16,24]. In one reaction setup, 80 mL of aqueous extract of *E. odoratum* was added to 400 mL of 1 mM AgNO_3_ and mixed on a magnetic stirrer. The ratio of aqueous extract of *E. odoratum* to AgNO_3_ in the reaction vessel was 1:5. In another reaction setup, 40 mL of aqueous extract of *E. odoratum* was added to 400 mL of 1 mM AgNO_3_ and mixed on a magnetic stirrer while heating at a temperature of about 90 °C. The ratio of aqueous extract of *E. odoratum* to AgNO_3_ in reaction vessel was 1:10. The final stage of reaction was marked with colour change and the appearance of localized surface plasmon resonance band in the UV-VIS spectrum. The resulting reaction mixtures were centrifuged to obtain the nanoparticles. 

### 2.4. Characterization of the Synthesized Nanoparticles

UV-VIS spectrophotometer (UV-1901UV-VIS spectrophotometer Agilent Technology, Cary series UV-VIS spectrometer, Santa Clara, CA, USA) was used to monitor the formation of the nanoparticles with absorbance in the range of 200–800 nm. Powdered X-ray diffraction (PXRD) spectra of the nanoparticles were obtained by an automated Röntgen PW3040/60 X’Pert Pro X-ray diffractometer (Almelo, Netherlands) with Ni-filtered Cu Kα radiation (λ = 1.5405 Å) at room temperature, in the 2θ range of 20°–90° with a scanning rate of 1 deg/min. X’Pert High Score Plus PW3212 software was used for the analysis of the PXRD results, and the phase identification was carried out using the Joint Committee on Powder Diffraction Standards (JCPDS). The surface morphology of the synthesized nanoparticles was observed by using Quanta FEG 250 Environmental scanning electron microscope (Hillsboro, OR, USA) under an acceleration voltage of 30 kV. Sample for TEM analysis was prepared by sonicating the sample for 30 min in water. A drop of the suspension was placed on carbon-coated copper grids. An extra sample was removed using blotting paper and the film on the grid was dried. The analysis was done using model JEOL2100 instrument (München, Germany) fitted with a LaB 6 electron gun, and images were captured using a Gatan Ultrascan digital camera. The shape and size of the nanoparticles was determined from TEM micrographs.

### 2.5. Mosquito Larvicidal Efficacy

#### 2.5.1. Collection of Mosquito Larvae

First and second instar larvae of *Culex quinquefasciatus* were collected from stagnant water within Abakaliki metropolis, Nigeria, and identified at the Entomology Unit, Department of Zoology, Ebonyi State University, Ebonyi State, Nigeria. The larvae were kept in different plastic trays containing tap water. They were reared in the laboratory to late third or early fourth instars with dog biscuits and yeast powder in a 3:1 ratio [46]. The third instar stage was collected after four days post-hatching, while the fourth instar stage was collected after six days post-hatching, with the larva reaching a length of almost 1/2 inch.

#### 2.5.2. Mosquito Larvicidal Assay

The synthesized nanoparticles were dissolved in 1 mL DMSO and prepared into different concentrations with double distilled water. Twenty-five reared third and fourth instar stages of *Culex quinquefasciatus* larvae were transferred by means of a dropper to 100 mL beakers. Mortality of the larvae was calculated at 12 and 24 h of exposure. During the exposure periods, no food was supplied to the larvae and the percentage of mortality was calculated. The experiment was performed in three replicates with the control under laboratory conditions at 28–30 °C. The data were presented as the mean ± standard deviation, and all the statistical analyses were performed by SPSS version 11.5. The percentage of mortality, LC50, LC90, and chi-squared test were calculated using Biostat Software Pro 5.9.8 [47].

#### 2.5.3. Dose Response Bioassay

Different concentrations (50, 100, 150, 200, and 250 ppm) of the nanoparticles were evaluated for the bioassay. The numbers of dead larvae were counted after 12 h and 24 h periods of exposure, and the percentage of mortality was reported from the average of three replicates.

Control mortality was corrected by using Abbott’s formula, and percentage mortality was calculated using Equation (1):

(1) $$\mathrm{Percentage Mortality}\;=\;\frac{\mathrm{Number of dead larvae}}{\mathrm{Number of larvae introduced}}\times 100$$

LC50 is the concentration of the sample is needed to eliminate 50% of the population of the mosquito larvae exposed to it, while LC90 is the concentration of the sample that will be needed to eliminate 90% of the population of the mosquito larva exposed to it.

### 2.6. Antimicrobial Studies

#### 2.6.1. Antimicrobial Assay

Clinical isolates of different microbial strains were collected from the Department of Microbiology, University College Hospital, Ibadan, Nigeria and were identified using various standard methods. The bacteria strains were Gram-negative, *Escherichia coli* (*E. coli*) and *Salmonella typhi* (*S. typhi*) *strains* and Gram-positive *Bacillus subtilis* (*B. Subtilis*) and *Staphylococcus aureus* (*S. aureus*) strains. The fungi organism was *Candida albicans.* The microbial strains were selected based on their clinical and pharmacological importance [48]. Antimicrobial screening was carried out using the agar disc diffusion method [49,50] and was carried out at the Department of Microbiology, University of Ibadan, Nigeria. 

The Petri plates were prepared using sterile Muller–Hinton agar (MHA). The inocula of test cultures (106 CFU/mL) were streaked on to the condensed Muller–Hinton agar in Petri plates by a sterilized cotton swab to ensure a uniform thick lawn or layer of growth and allowed to dry for 15 min. The concentration (100 μg/mL) of the nanoparticle stock solutions was prepared using 100% dimethyl sulfoxide as diluent [51]. The stock solutions of the plant extract (EO) and standard drugs were prepared using double distilled water to the same concentration as the nanoparticles. Sample dilutions were performed as described by the Clinical and Laboratory Standards Institute (CLSI) (2002) [52]. Blank paper disks with a diameter of 6.0 mm were impregnated with 25 μL of the nanoparticles stock solution. To each plate 20 μL of 1.25 mg/mL 3-(4,5-dimethylthiazol-2-yl)-2,5-diphenyltetrazolium bromide (MTT) (Sigma-Aldrich, St. Louis, MO, USA) was added and observed for a purple colouration after incubation at 37 °C for 30 min, which indicated microbial growth [53]. The plates were incubated for 24 h at 37 °C for the bacteria and for 48 h at 30 °C for the fungi. Control experiments were carried out under similar conditions by using a commercially-available antibacterial drug (Streptomycin) and an antifungal drug (Ketoconazole) as the positive control drugs, while 100% dimethylsulfoxide was used as a negative control. The sensitivities of the microorganism species to the samples were determined by measuring the sizes of inhibitory zones (including the diameter of disk) on the agar surface around the disks, and values <6 mm were considered as not active against microorganisms. Zones of inhibition were recorded in millimetres and the experiment was repeated twice. Experimental results were given as mean ± S.D. of the two parallel measurements. Analysis of variance was performed by ANOVA procedures. Significant differences between means were determined by Duncan’s multiple range tests. *p* values of <0.05 were regarded as significant. 

#### 2.6.2. Minimum Inhibitory Concentration (MIC) Studies for the Nanoparticles

A minimum inhibitory concentration study for the nanoparticles was performed using the broth dilution method [54]. This was determined using a 96-well microtitre plate format. Different concentrations within the range 15–100 μg/mL from the 100 μg/mL stock solutions of silver nanoparticles was prepared with DMSO as a diluent. The inocula were prepared by making a direct broth suspension of isolated microbial colonies and adjusting them to achieve a turbidity equivalent to a 0.5 McFarland turbidity standard. The suspension in the tube finally contained 10^6^ CFU/mL of the test organisms. Each well of the microtitre plate (96 wells) were filled with 100 μL of nutrient broth, 20 μL of 10^6^ CFU/mL of the test organisms, and 80 μL of different concentrations of the nanoparticles ranging from 15–100 μg/mL. Control wells were filled with broth and test organisms only. After 24 h incubation at 37 °C for the bacterial strains and 48–72 h at 30 °C for the fungi organisms, 20 μL of 1.25 mg/mL 3-(4,5-dimethylthiazol-2-yl)-2,5-diphenyltetrazolium bromide (MTT) (Sigma-Aldrich) was added to each well and observed for a purple colouration after incubation at 37 °C for 30 min, which indicated microbial growth [51]. The minimum concentrations of the nanoparticles that did not show any colour change in the wells were recorded as the MIC. The method was replicated three times to validate the results.

## 3. Results and Discussion

### 3.1. UV-VISible Spectral Studies

**Figure 1:**
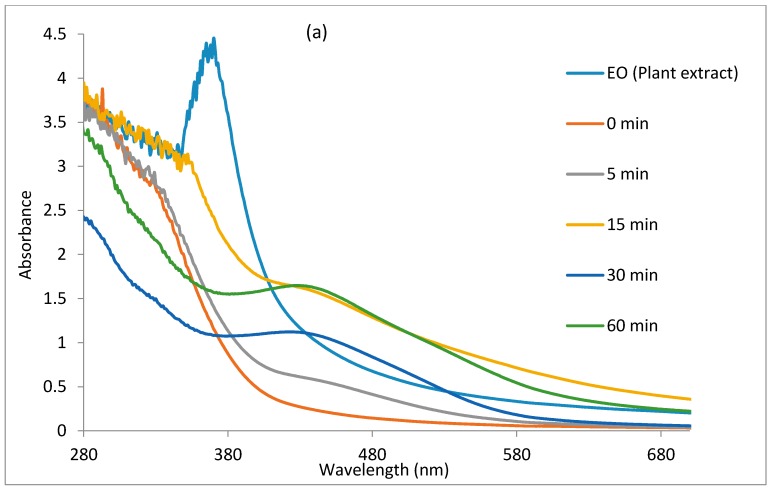

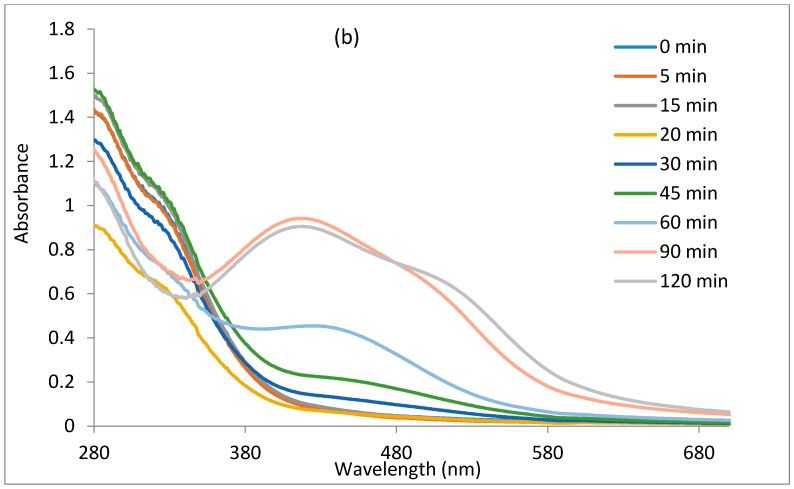
The UV-VIS spectra of the nanoparticles using plant leaf extract as the reducing agent: (**a**) the concentration of plant leaf extract to AgNO_3_ at a ratio of 1:5; and (**b**) the concentration of plant leaf extract to AgNO_3_ at a ratio of 1:10.

The reduction of Ag^+^ ions to Ag^0^ by *E. odoratum* leaf extract was monitored with the use of a UV-VIS spectrophotometer by recording the absorption as a function of time, as shown in Figure 1a,b. Figure 1a shows the UV-VIS spectra of the nanoparticles synthesized at a 1:5 ratio of plant extract to silver nitrate, while Figure 1b shows the UV-VIS spectra of silver nanoparticles synthesized at a 1:10 ratio of plant extract to silver nitrate. The figures show that an increase in contact time increases the intensity of the localized surface plasmon resonance, reflecting an increase in the concentration of nanoparticles up to a point before the concentration of nanoparticles decreases. In Figure 1a, the nucleation appears to proceed faster compared to Figure 1b, as localised surface plasmon resonance bands were noticed at 30 min of the reaction period, while it became visible after 60 min in Figure 1b. The localised surface plasmon resonance band showed maximum absorbance at 424 nm in Figure 1a. In Figure 1b, the maximum absorbance band of the localised surface plasmon resonance appeared at 424 nm after 60 min and exhibited a blueshift to 418 nm after 120 min, but with a reduction in intensity and appearance of a broad plasmon band and a hump around 515 nm. The reduction in intensity of the band in Figure 1b may be attributed to some digestive ripening among the nanoparticles which will saturate the reaction with polydispersed nanoparticles of smaller sizes and equally larger ones, hence, the observed band around 515 nm [55,56]. The optical absorption spectra of metal nanoparticles is dominated by the SPR, which shifts toward the red or blue end depending upon the particle size, shape, state of aggregation, and the surrounding dielectric medium [57,58].

### 3.2. Transmission Electron Microscopy (TEM) Analysis

**Figure 2:**
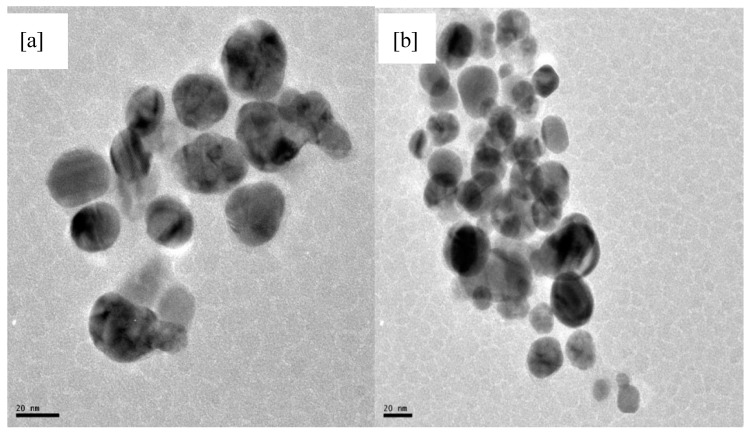
Transmission electron microscopy image of the nanoparticles after 120 min synthesis time: (**a**) the concentration of the 1:5 extract to AgNO_3_ ratio; and (**b**) the concentration of the 1:10 extract to AgNO_3_ ratio.

Transmission electron microscopy (TEM) was employed to determine the size, shape, and particle morphology of the synthesized nanoparticles. The representative TEM micrographs are shown in Figure 2a,b. Figure 2a showed that the morphology of the nanoparticles was spherical and with an average particle size of 23.6 nm. The dispersed appearance of the nanoparticles indicates stabilization by some capping agents. The effect could be due to capping by saponins and proteins. In Figure 2b, there seems to be digestive ripening of the larger molecules into smaller particles, which culminated in agglomeration and polydispersity [42]. This was observed when the concentration ratio of the extract to AgNO_3_ was reduced (i.e., 1:10). The particles size is in the range of 8.2–20.5 nm. The figure depicts particles of varied sizes, which was supported by the nature of the plasmon bands shown in Figure 1b.

### 3.3. Powder X-ray Diffraction (PXRD) Results

**Figure 3:**
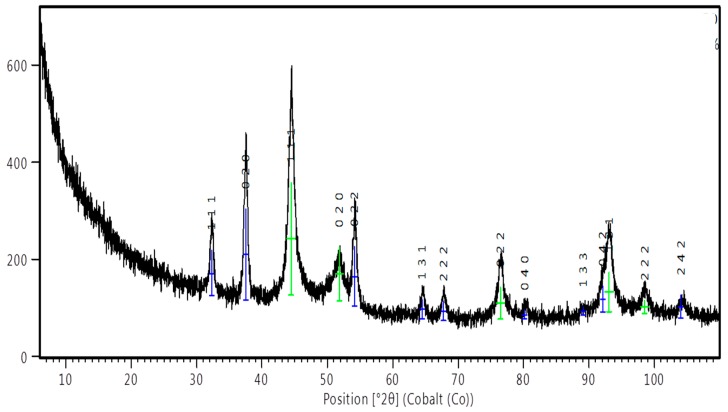
Representative PXRD patterns for the synthesized nanoparticles (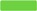 silver, 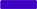 silver oxide).

**Scheme 1:**
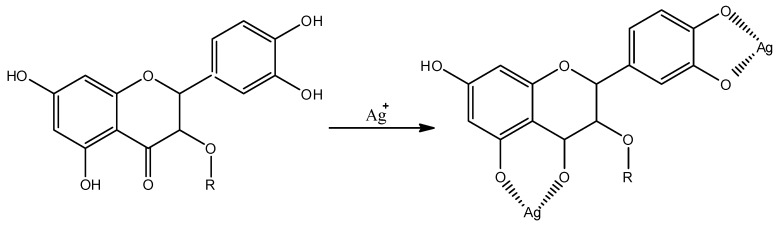
Proposed molecular binding of flavonoids (in EO) with silver ions during the reaction [59].

Representative PXRD patterns for the synthesized nanoparticles is presented in Figure 3. The peaks at 2θ of 44.48°, 51.84°, 76.43°, 93.04°, and 98.56° are related to the (111), (020), (022), (131), and (222) planes of Ag, which matches with the reflections of crystallographic planes of the face-centered cubic structure of metallic silver (JCPDS file No. 04-004-6436). The (111) orientation of the silver is more intense than other peaks suggesting (111) as its predominant orientation. The pattern also shows peaks at 2θ of 32.30°, 37.49°, 54.11°, 64,48°, 67.73°, 80.13°, 89.09°, 92.07°, and 104.10°, which are attributed to (111), (020), (022), (131), (222), (040), (133), (042), and (242) planes of Ag_2_O nanocrystals (JCPDS No. 04-006-5378). Co-existence of silver and silver oxide nanoparticles synthesized by extracts of plants has been reported [59]. Plant extracts contain various organic compounds (e.g., flavonoids) that possess various oxygen-containing functional groups that may coordinate with silver ions to form coordination bonds due to their ability to donate electrons [59] as shown in Scheme 1. This process may be responsible for the oxidation of silver to silver oxide. In addition, the peak broadening of Ag and Ag_2_O suggests that the typical size of the particles is small, and this is consistent with our TEM observation.

### 3.4. Mosquito Larvicidal Studies

The larvicidal activity of the nanoparticles were compared to that of aqueous leaf extract of *E. odoratum* and their effective larvicidal activities against third and fourth instar larvae of *Cx. quinquefasciatus* after 12 and 24 h exposure times are presented in Table 1. The *E. odoratum* leaf extract was slightly toxic against the larval instars (III and IV) of *Cx. quinquefasciatus*. The LC50 and LC90 values obtained at 12 h exposure period on III larval instar of *Cx. quinquefasciatus* were 461.6 ppm and 807.6 ppm respectively and at 24 h exposure time were 396.8 ppm and 716.8 ppm respectively. Also, the LC50 and LC90 values obtained at 12 h exposure period on IV larval instar of *Cx. quinquefasciatus* were 553.4 ppm and 953.4 ppm, respectively, and at 24 h exposure time, 448.3 ppm and 803.9 ppm, respectively. A dose-dependent effect was found in the study, and were also reported for a growing number of botanicals [60,61]. The nanoparticles were found to be very toxic against third and fourth instar of *Cx. quinquefasciatus* larvae. The LC50 and LC90 values obtained on testing against III larval instars of *Cx. quinquefasciatus* were 148.5 ppm and 420.9 ppm, respectively, at 12 h of exposure, and LC50 and LC90 of 95.9 ppm and 337.5 ppm, respectively, at 24 h of exposure. Additionally, the LC50 and LC90 values obtained on testing against IV larval instars of *Cx. quinquefasciatus* were 217.1 ppm and 483.8 ppm, respectively, at 12 h of exposure and 166.383 ppm and 438.723 ppm, respectively, at 24 h of exposure. Increasing the exposure time from 12 h to 24 h has a significant effect on the mortality of mosquito larvae. Similarly, the effect of increasing larvicide concentration is significant on the mortality of mosquito larvae. Furthermore, higher toxicity was obtained testing the nanoparticles compared to the extracts of EO, since LC50 and LC90 values are lower than that of the plant extracts. The reports were similar to a growing number of green-fabricated AgNPs that have been reported as effective pesticides against different mosquito vectors of medical and veterinary importance [17,20]. Vector control is an important aspect of public health, mostly as it concerns mosquito-borne diseases. Hence, the invention of nanoparticles using *E. odoratum* leaf extract, as represented here, could provide a new product to prevent the proliferation of mosquito-borne diseases and replace synthetic larvicidal products.

The toxicity to the late third instar larvae of *Culex quinquefasciatus* by methanolic leaf extract of *Memordica charantia*, *Trichosanthus anguina, Luffa acutangula, Benincasa cerifera*, and *Citrullus vulgaris* showed the LC50 values of 465.85, 567.81, 839.81, 1189.30, and 1636.04 ppm, respectively [62]. The present study clearly proved the bioefficacy of *E. odoratum* extracts on *C. quinquefasciatus* since we obtained lower L50 at 12 h of exposure against third instars of *C. quinquefasciatus*. *E. odoratum* extracts might lead to better applications of botanical derivatives during the suitable developmental period and could also be helpful as a natural mosquitocide which, in the future, might be used directly as a larvicidal agent in small-volume aquatic habitats or breeding sites of limited size around human dwellings.

### 3.5. Antimicrobial Analysis 

**Figure 4:**
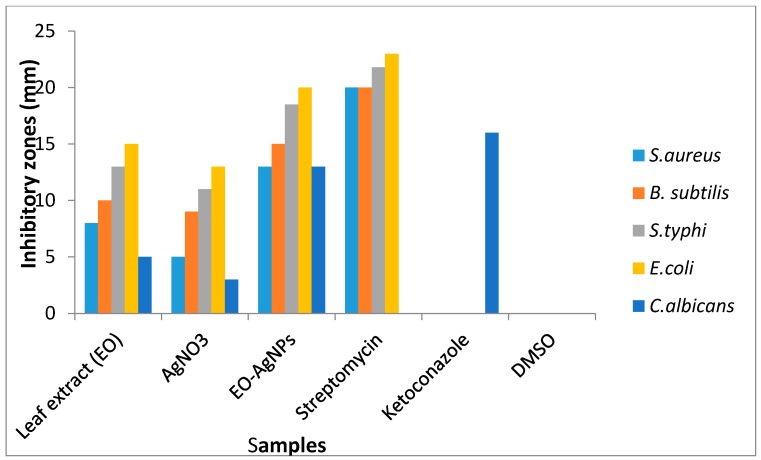
Zones of inhibition of the samples against some micro-organisms.

Antimicrobial evaluation of the nanoparticles was compared to the antimicrobial results of EO plant extract, AgNO_3_, and standard drugs. The results are presented as a histogram in Figure 4. The antimicrobial activities of the samples were found to be, in decreasing order, NPs ˃ AgNO_3_ ˃ EO plant extract. The plant extracts (EO) gave antimicrobial activities with inhibition zones in the range 8–15 mm while the AgNO_3_ gave inhibition zones in the range 5–13 mm. The nanoparticles exhibited good antimicrobial activities with inhibition zones in the range of 13–20 mm. The NPs showed the best antimicrobial activities among the screened samples. They were more active against the bacterial strains than the fungal organism. In the case of *E. coli* and *S. typhi*, the nanoparticles gave 87% and 85% antibacterial activities, respectively, compared to that of the standard drug, streptomycin. Similarly, the bactericidal activities of the nanoparticles were higher for Gram-negative bacteria compared to Gram-positive bacteria. The observed increased antibacterial properties of the nanoparticles against Gram-negative bacteria strains compared to Gram-positive bacteria strains could be attributed to the difference in lipophilicity of the nanoparticles through the different bacteria cell compositions. Gram-positive bacteria contain a rigid cell wall network with a peptidoglycan layer making it resistant to mechanical rupture, while Gram-negative bacteria have a cell membrane network that is only one molecule thick, together with up to 25% (mass) of lipoprotein and lipopolysaccharide. The miniature size of nanoparticles has been implicated as supporting permeability through bacteria cells. However, the more fortified cell wall of the Gram-positive bacteria makes it less penetrable compared to the cell membrane of the Gram-negative bacteria [63,64]. The nanoparticles also gave the best antifungal activity compared to the plant extract and AgNO_3_. The results of the antimicrobial screening of the silver nanoparticles are in agreement with other reported works on green-mediated silver nanoparticles [60,65]. The antimicrobial effect of each dose of the samples was calculated statistically. The regression line and estimated value with standard error were also considered. All of the experiments were performed thrice. However, this experimental data was further investigated by applying the ANOVA test (*p* < 0.05). Results show that about 99.8% of the variances were accounted for because the value of the adjusted R^2^ is 0.998.

The plant extracts and silver nitrate showed no activity against all of the microbes below the concentration of 100 μg/mL concentration which was used for the agar diffusion experiment, hence, their antimicrobial activity could not be evaluated beyond that concentration (MIC). However, the silver nanoparticles were evaluated for their MIC having shown activity below the concentration of 100μg/mL. The minimum inhibitory concentration gives information on the minimum concentration in which a sample will inhibit the growth of a microbial strain. Hence, the lower the minimum inhibitory concentration of the compound against a microbe, the higher is its antimicrobial efficacy towards the microbial strains. MIC was carried out on the NPs due to the large microbial inhibition obtained compared to leaf extract and the silver nitrate at a concentration of 100 μg/mL against the pathogens (Figure 4). The MIC values of the NPs were 25 μg/mL for *E. coli*; 40 μg/mL for *S. typhi*; 100 μg/mL for *C. albicans*, and 75 μg/mL for both *B. subtilis* and *S. aureus* (Table 2). The lowest minimum inhibitory concentration for NPs was towards *E. coli* and considered to be most active toward the bacteria strain.

With MIC results below the concentration of 100 μg/mL against the bacterial strains for the nanoparticles, compared to the antibacterial results of plant extracts and silver nitrate at a concentration of 100 μg/mL, the nanoparticles are considered the best antibiotic among the samples. However, the MIC result of the nanoparticles is the same with that of the plant extract and silver nitrate, hence, the result obtained from the agar diffusion experiment becomes the only established antibacterial activity for the samples. 

Although, in general, the microbial drugs showed the most antimicrobial activity in the entire test samples, the nanoparticles showed 86.95% and 92.5% of the antibacterial activities of streptomycin against *E. coli* and *S. typhi*, respectively. Similarly, the nanoparticles gave 81.25% of the antifungal activity of ketoconazole against *C. albicans*.

## 4. Conclusions

This research focused on eco-friendly nano-synthetic routes to mosquitocidal and antimicrobial NPs. UV-VIS, FTIR spectroscopic measurements, XRD, and TEM analysis showed evidence of the rapid and cheap synthesis of Ag/Ag_2_O nanoparticles. The nanoparticles were spherically shaped and stabilised by the constituents of *E. odoratum* leaf extracts. The biological applications of the nanoparticles showed that they have high larvicidal efficacy against III and IV instar larvae of *Cx. quinquefasciatus* and also exhibited broad-spectrum antimicrobial potential against different microbial strains. Our results pointed out that the Ag/Ag_2_O nanoparticles can be proposed as effective tools to reduce larval populations of *Cx. quinquefasciatus* and as an antibiotic. However, extensive field assays based on the use of plant-borne metabolite-capped metal nanoparticles against mosquito vectors and different microbes will be carried out in the future to further substantiate this claim.

## Figures and Tables

**Table 1:** Larvicidal activity of the nanoparticles and EO aqueous leaf extract against *Culex quinquefasciatus* larvae.

Sample	Larvae Instar	Conc. (ppm)	Exposure Time (h)	%Mortality ± Standard Error	LC50 (ppm)	LC90 (ppm)	χ^2^ (d = 4) (n.s.)
EO-AgNPs	III	50	12	34.67 ± 0.33	148.5	420.9	0.017
		100		40.00 ± 0.58			
		150		46.67 ± 0.33			
		200		58.67 ± 0.33			
		250		70.67 ± 0.33			
		50	24	42.67 ± 0.33	95.9	337.5	0.983
		100		49.33 ± 0.33			
		150		60.00 ± 1.16			
		200		68.00 ± 1.00			
		250		81.33 ± 0.88			
	IV	50	12	25.32 ± 0.67	217.1	483.8	0.585
		100		25.33 ± 0.33			
		150		33.33 ± 0.67			
		200		46.67 ± 0.67			
		250		60.00 ± 1.16			
		50	24	33.33 ± 0.33	166.4	438.7	1.111
		100		34.67 ± 0.88			
		150		45.33 ± 0.88			
		200		49.33 ± 0.33			
		250		70.67 ± 0.88			
Plant Extract (EO)	III	50	12	6.67 ± 0.33	461.6	807.6	0.013
		100		9.33 ± 0.33			
		150		12.00 ± 0.00			
		200		14.67 ± 0.33			
		250		24.00 ± 0.58			
		50	24	8.00 ± 0.58	396.8	716.8	0.781
		100		13.33 ± 0.33			
		150		16.00 ± 0.00			
		200		17.33 ± 0.33			
		250		30.67 ± 0.33			
	IV	50	12	5.32 ± 0.33	553.4	953.4	0.612
		100		8.00 ± 0.00			
		150		9.32 ± 0.33			
		200		12.00 ± 0.00			
		250		17.33 ± 0.67			
		50	24	9.33 ± 0.33	448.3	803.9	5.162
		100		9.33 ± 0.67			
		150		12.00 ± 0.00			
		200		17.33 ± 0.67			
		250		26.67 ± 0.33			

No mortality was observed in the control. LC50 = lethal concentration (ppm) that kills 50% of the exposed organisms; LC90 = lethal concentration (ppm) that kills 90% of the exposed organisms; χ^2^ = chi-square value; df = degrees of freedom; n.s. = not significant (*p* ˂ 0.05).

**Table 2:** MIC of the nanoparticles (Ag/Ag_2_ONPs).

Microbial Strains	MIC
*Staphylococcus aureus*	75 μg/mL
*Bacillus subtilis*	75 μg/mL
*Salmonella typhi*	40 μg/mL
*Escherichia coli*	25 μg/mL
*Candida albicans*	100 μg/mL

μg = microgram.
